# Determinants of intra-household food allocation between adults in South Asia – a systematic review

**DOI:** 10.1186/s12939-017-0603-1

**Published:** 2017-06-21

**Authors:** Helen Harris-Fry, Niva Shrestha, Anthony Costello, Naomi M. Saville

**Affiliations:** 1 London School of Hygiene and Tropical Medicine, Keppel Street, London, WC1E 7HT UK; 2 Maternal Child and Adolescent Health, World Health Organization, Geneva, Switzerland; 3 Institute for Global Health, University College London, 30 Guilford Street, London, WC1N 1EH UK

**Keywords:** Food allocation, Equity, Intra-household, South Asia, Food habits, Gender, Systematic review

## Abstract

**Background:**

Nutrition interventions, often delivered at the household level, could increase their efficiency by channelling resources towards pregnant or lactating women, instead of leaving resources to be disproportionately allocated to traditionally favoured men. However, understanding of how to design targeted nutrition programs is limited by a lack of understanding of the factors affecting the intra-household allocation of food.

**Methods:**

We systematically reviewed literature on the factors affecting the allocation of food to adults in South Asian households (in Afghanistan, Bangladesh, Bhutan, India, Islamic Republic of Iran, Maldives, Nepal, Pakistan, Sri Lanka) and developed a framework of food allocation determinants. Two reviewers independently searched and filtered results from PubMed, Web of Knowledge and Scopus databases by using pre-defined search terms and hand-searching the references from selected papers. Determinants were extracted, categorised into a framework, and narratively described. We used adapted Downs and Black and Critical Appraisal Skills Programme checklists to assess the quality of evidence.

**Results:**

Out of 6928 retrieved studies we found 60 relevant results. Recent, high quality evidence was limited and mainly from Bangladesh, India and Nepal. There were no results from Iran, Afghanistan, Maldives, or Bhutan. At the intra-household level, food allocation was determined by relative differences in household members’ income, bargaining power, food behaviours, social status, tastes and preferences, and interpersonal relationships. Household-level determinants included wealth, food security, occupation, land ownership, household size, religion / ethnicity / caste, education, and nutrition knowledge. In general, the highest inequity occurred in households experiencing severe or unexpected food insecurity, and also in better-off, high caste households, whereas poorer, low caste but not severely food insecure households were more equitable. Food allocation also varied regionally and seasonally.

**Conclusion:**

Program benefits may be differentially distributed within households of different socioeconomic status, and targeting of nutrition programs might be improved by influencing determinants that are amenable to change, such as food security, women’s employment, or nutrition knowledge. Longitudinal studies in different settings could unravel causal effects. Conclusions are not generalizable to the whole South Asian region, and research is needed in many countries.

## Background

Every day, households must make difficult decisions about how limited food should be shared among their members. In low-income countries, around 793 million people are undernourished [1] and over 3.5 million mothers and children under five die every year because they are undernourished [2]. So, these food allocation decisions have important nutritional, and sometimes life-critical, consequences.

It is often assumed that food is allocated inequitably in households in the United Nations-defined region of South Asia (in Afghanistan, Bangladesh, Bhutan, India, Islamic Republic of Iran, Maldives, Nepal, Pakistan, Sri Lanka) [3]. Tangential evidence of significantly higher female than male infant mortality rates [4] and social and cultural gender discrimination that pervades numerous cultural and religious practices [5, 6] are suggestive of a plausible pathway by which discrimination against women leads to them not receiving their ‘fair share’ of food [7]. For instance, young women often stay at home and avoid moving around or interacting with the community (a practice known as *purdah*), women often describe their husband as their God, and it is common for women to serve men first and themselves last [8, 9].

International reviews have not found a consistent global trend of inequitable intra-household food allocation, except in South Asia [10–12]. In South Asia the scant evidence available suggests that women are discriminated against and receive less than their ‘fair share’, particularly in the allocation of high status, nutrient-rich foods [9, 13, 14]. This means that nutrition programs providing social transfers at the household level may fail to reach the intended target recipients, such as the most undernourished or pregnant women. On the other hand, if program implementers know which factors affect food allocation, and can identify those amenable to change, programs could be designed to target those individuals more effectively. Furthermore, behaviour change interventions without social transfers, or other programs or policies not directly related to nutrition, may be able to increase intra-household equity and improve nutritional outcomes by pushing the right levers.

However, intra-household food allocation has often been described as a ‘black box’ that is poorly understood [15, 16]. This may be because economic consumption and nutrition surveys are typically collected at the household rather than individual level, and also because evidence has been segregated by academic discipline. Thus, this study aims to identify the determinants of intra-household food allocation, focusing on allocation between adults from South Asian households, using a systematic and multidisciplinary literature review.

## Materials and methods

We followed guidelines on systematic search protocols from Reeves et al. [17] and Petticrew and Roberts [18] and reporting guidelines (Preferred Reporting Items for Systematic Reviews and Meta-Analyses (PRISMA) [19]) when relevant.

### Search method

In two phases between October 2015 and January 2017, two authors independently ran and filtered the searches in PubMed, Web of Science Core Collection, and Scopus databases. The former two databases were included because they contain multi-disciplinary peer-reviewed literature, and the latter database (Scopus) because it contains non peer-reviewed literature.

We used a combination of medical subject heading (MeSH) and free text search terms, using asterisks to indicate a wildcard operator. The search syntax used in PubMed was:

(((((family OR household*)) AND (food OR energy intake OR food habits OR diet OR nutrition*)) AND (allocat* OR distribut* OR decision* OR shared OR sharing OR share))) AND (age factors OR "age" OR sex OR "gender")

South Asia was not used as a search term because databases have different systems for cataloguing studies geographically, and because international or theoretical results were also included. Instead, studies from other regions were excluded in the filtering process. This increased the sensitivity of the search at the expense of specificity, giving more irrelevant (but also more relevant) results. Other search terms relating to ‘inequity’, and ‘determinants’ were also not included to ensure adequate sensitivity.

### Inclusion and exclusion criteria

We included quantitative, qualitative, anthropological, and theoretical studies from peer-reviewed and non-peer-reviewed sources that referred to the determinants of inequity in intra-household food allocation among adults. Inequity was defined as occurring when one person’s food needs were met more adequately than another’s needs [12]. ‘Food’ could refer to calories, nutrients, food quantities, food types, or dietary diversity, and ‘needs’ were defined as biological requirements. We also included any papers that described the determinants of inequitable food allocation without explicitly measuring food intakes or needs if the paper referred to *relative food allocations within households,* rather than effects on absolute intakes.

Recognising that the capability to control food distribution decisions might be as important for wellbeing outcomes as the resulting food allocation [20, 21], we considered including literature on the determinants of control over food selection, preparation, and serving. However, since this review is intended to inform nutrition programming, and to limit the scope, we focussed on food allocation outcomes only. Therefore, this review excluded the extensive and multidisciplinary body of work on intra-household influences on food purchasing and preparation decisions, unless they were shown to affect the distribution of food.

We included any studies explaining inequity between different adults (different age-sex groups or different pregnancy or lactating status). The cut-off for adults was ≥15 years because studies often include ‘women of reproductive age’ by using the age range of 15 to 49 years. We excluded papers that only referred to food allocation among children (or between adults and children), or did not report on food allocation directly, for example by referring to anthropometry or energy expenditure. We also excluded any papers that did not refer to any determinants other than age-sex group or pregnancy status.

We included all studies relating to the UN-defined region of South Asia: Afghanistan, Bangladesh, Bhutan, India, Islamic Republic of Iran, Maldives, Nepal, Pakistan, Sri Lanka. We also included theoretical and international references that were not specific to a particular research setting. There was no publication date cut-off, because we predicted that empirical and high quality evidence would be limited and so did not want to exclude dated but relevant literature.

The search results were exported into EndNote reference management software for filtering. Excluded papers were organised into different folders labelled according to their reason for exclusion. Disparities in inclusion / exclusion decisions were resolved by consultation with a third author. From the systematic search results, we searched through the reference lists to find additional relevant results. We added additional sources by communication with the authors.

### Study quality assessment

To assess the quality of quantitative results we used a modified Downs and Black checklist [22], and for qualitative results we used the Critical Appraisal Skills Programme checklist [23]. Given the breadth and heterogeneity of results expected, a meta-analysis was not possible and so sources of bias were not factored into the data synthesis.

### Data extraction strategy

Two authors independently extracted the author name, publication year, study location, study method, and determinant(s) of food allocation into Microsoft Excel databases. We then mapped out each determinant on paper, by grouping them into different themes. The identified themes were discussed and disparities resolved by referring back to the text. These themes were compiled into a conceptual framework, with discussion from all authors.

## Results

**Fig. 1:**
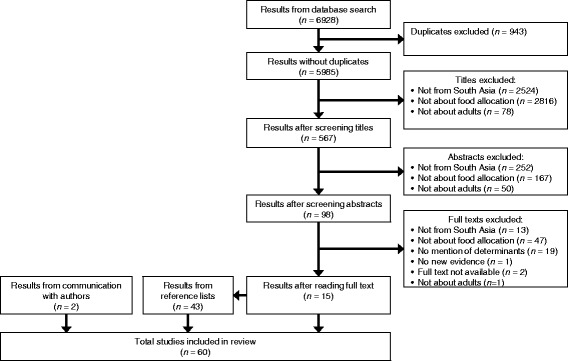
The exclusion of database results and inclusion of results from references and communication with authors

The result filtering process is shown in Fig. 1. Fifteen results were identified from the database search, 43 results were added by searching references, and two papers were added from communication with the authors, giving a total of 60 results.

The publication date, location, study and analysis method, sample size and characteristics, determinants, and food allocation outcome measures for the selected studies are summarised in Tables 1, 2 and 3. Of the 60 studies reviewed, around one quarter quantitatively estimated the associations between at least one determinant and food allocation, another quarter were qualitative studies, and the remaining half made theoretical, anecdotal, or speculative references to determinants. Nearly every quantitative study used a different outcome measure, whereas the qualitative, theoretical and anecdotal results tended not to define the outcome or discuss differences in nutritional requirements. Over half were from Bangladesh (*n* = 14) or India (*n* = 18), and around one third did not refer to any specific country or were international reviews. Publication dates ranged between 1972 and 2016, and 70% were published before 2000.

**Table 1:** Quantitative studies: Geographical and methodological characteristics of selected articles (*n*=16)

Author	Year	Study method	Sample size	Sample characteristics	Analysis method	Determinant	IHFA outcome
Bangladesh (*n* = 5)							
Abdullah and Wheeler [65]	1985	Longitudinal 4x 3 day WFR	53 HH	Rural Muslim households with at least one child under 5 years, from one village. Men and non-pregnant, non-lactating women (age not specified)	Analysis of variance	Season (March to July vs September to December)	RDEAR = Relative Dietary Energy Adequacy Ratio (individual calorie intake as a proportion of body weight / adult male calorie intake as a proportion of body weight)
Bouis and Novenario-Reese [69]	1997	Longitudinal 2x 1 day WFR	590 HH	Households from 8 rural *thanas.* Men and women aged >18 years (average age 39 and 35 years respectively)	Regression (coefficients not reported)	• Occupation (farmer or agricultural labourer) • Age and education of head of household • Land ownership	FS/ES = Ratio between ‘food share’ (FS), proportion of total household food that a person consumed, and ‘energy share’ (ES), proportion of household calories that an individual consumed.
Kumar and Bhattarai [61]	1993	Longitudinal 3x 1 day WFR	300 HH	Households from 8 villages in 4 districts. Men and women aged >18 years	Multivariate analysis (more detail not given; results described but effect size not reported)	Household caloric adequacy	Calorie ‘adequacy’ (Intakes / Requirements)
Pitt, Rosenzweig and Hassan [32]	1990	Longitudinal 1x 1 day WFR in 335 HH; 4x 1 day WFR in 50 HH	385 HH	Bengali households from 15 villages (excludes hill tribes). Men and women aged ≥12 years.	Linear regression coefficient	Health endowments	Calorie intake
Tetens et al. [72]	2003	Longitudinal 2x 1 day WFR	304 HH	Two rural villages in lean and peak seasons. Men and women aged 18 to <30, 30 to <60, and ≥60.	Analysis of variance	• Season (lean vs peak season) • Village • Socio-economic status	Calorie intake
India (*n* = 8)							
Aurino [37]	2016	Longitudinal 2x 1 day survey (older cohort only)	976 HH	20 clusters, with over-sampling in disadvantaged areas. >90% Hindu, and 8% female headed households. Older cohort of boys and girls includes adolescents aged 15 years.	Linear regression coefficient	• Puberty (growth) • School enrolment • Time use • Number of meals • Physical activity	Dietary Diversity Score by gender
Babu, Thirumaran and Mohanam [62]	1993	Longitudinal 6x 3 day WFR	120 HH	1 rural village in peak and lean seasons. Sample includes non-agricultural workers (mainly silk weavers), agricultural labourers, and land owning subsistence or ‘market-oriented’ cultivators. Men and women (age not specified)	Descriptive comparisons	• Season • Occupation (subsistence, market-oriented, non-agricultural, and agricultural labourer households)	RDEAR = Relative Dietary Energy Adequacy Ratio (Individual calorie intake as a proportion of individual requirements / Adult male intake as a proportion of his requirements); RDPAR = Relative Dietary Protein Adequacy Ratio (Individual protein intake as a proportion of requirements / Adult male intake as a proportion of his requirements)
Barker et al. [45]	2006	Cross-sectional 1x 1 day survey	101 HH	1 rural village, mostly cash crop farmers. Selected households containing a minimum of: husband and wife (age not specified), plus son and daughter both aged 3 to 8 years.	Principal component analysis	• Farm work, household chores	Oil intake (g), and frequency of snacking, fasting, and missing meals
Basu et al. [67]	1986	Cross-sectional 1x 1 day 24h	219 HH	Households from West Bengal, with men and women aged > 18 years.	Analysis of variance	• Rural vs urban • Occupation (agriculturalist vs plantation worker) • Religion • Ethnicity • Microeconomic subgroups	EI-ER (Energy intake - Energy requirements), and age-sex groups ranked in order of EI-ER
Behrman and Deolalikar [63]	1990	Longitudinal 4x 1 day 24h	2 rounds of 120 HH	Three rural villages. Sampling stratified to include landless agricultural labourers and landowning cultivators. Men and women (age group not specified)	Linear regression coefficient	Food price elasticities	NAR = Nutrient adequacy ratio (Nutrient intakes / Requirements)
Brahmam, Sastry and Rao [66]	1988	Cross-sectional 1x 1 day 24h	1878 HH	10 Indian states, selected households with at least one member of preschool age.	Descriptive comparison for adults	Household calorie adequacy (based on intakes of all respondents within the household)	Calorie adequacy (‘adequate’ = Calorie intake ≥ 70% Recommended Daily Intakes)
Chakrabarty [73]	1996	Longitudinal 2x 2 day 24h	221 HH	Three groups (high caste, Scheduled Tribe, Scheduled Caste) in West Bengal. Sampled nuclear families with both parents alive, non-working women (for high caste) and working women (for Scheduled Tribe).	*t*-test	Availability of food (lean vs peak season)	Cereal intake – Recommended cereal intakes for a balanced diet
Harriss-White [27]	1991	Longitudinal 4x 1 day 24h	176 HH	Six villages in central and southern India. Men and women (age not specified)	*t*-test	• Season • Region • Land holding vs landless	RI = Relative calorie intakes (Individual intakes / Adult male intakes)
Nepal (*n* = 1)							
Gittelsohn [9]	1991	Cross-sectional 1x 1 day 24h & observation	115 HH	Six villages in Western hills. Men and women aged 18-24, 25-49, and ≥50	Correlation	Food serving habits, including serving order, asking for food, having second helpings, substituting foods, and channelling foods.	FQS = Food quantity score (individual consumption as a proportion of total household consumption / Individual body weight as a proportion of total household body weight)
Pakistan (*n* = 1)							
Government of Pakistan [38]	1979	Cross-sectional 1x 24h	975 HH	Male head of household, plus woman of childbearing age (preferably pregnant or lactating) and all children aged under 3 years.	Linear regression (coefficients not reported)	• Education • Region • Household size • Income	Individual intake / Household intake (calories, protein, iron and vitamin A)
Sri Lanka (*n* = 1)							
Rathnayake and Weerahewa [30]	2002	Cross-sectional 1x 24h	60 HH	Households from lower income group in urban Kandy. Fathers and mothers (age not specified)	Linear regression coefficient and *t*-test	• Mother’s income • Mother’s education • Ethnicity • Family size	RCA = Relative calorie allocation (calorie intake as a proportion of recommended allowance / Household intake as a proportion of household allowance)

*WFR* Weighed food records, *24h* 24-hour dietary recall, *HH* Households

**Table 2:** Qualitative studies: Geographical and methodological characteristics of selected articles (*n*=15)

Qualitative studies (*n* = 15)							
Author	Year	Study method	Sample size	Sample characteristics	Analysis method	Determinant / theme	IHFA outcome
Bangladesh (*n* = 4)							
Abdullah [36]	1983	Unstructured interviews	40 HH	One rural Muslim village in central-west Bangladesh. Mostly male respondents. Particularly in poor households, women also participated.	Notes recorded on paper, and results analysed by wealth group.	• Economic contributions • Norms relating to receiving a ‘fair share • Food security and scarcity • Household structure (allocation to women in parental vs marital homes)	Food allocation
Mukherjee [75]	2002	Seasonal calendar	1 group	Not reported	Method of quantifying discrimination not specified	• Season	Discrimination in consumption of food items and types
Naved [35]	2000	Focus group discussions, case studies, and other methods.	Case studies of 22 women; 19 men	Three villages participating in an agricultural program. Male and female beneficiaries of the program.	Triangulation of multiple qualitative techniques	• Physically strenuous labour contributions • Bargaining power • Individuals tastes and preferences (women eating more less-preferred foods than men) • Food availability (Seemingly contradictory anecdotes that increased food availability did not change food allocation patterns much, but food scarcity led to men being less likely to have sufficient food than women.)	Allocation of specific food items
Rohner and Chaki-Sircar [71]	1988	Observation? (*limited detail*)	1 village	Not reported	Not reported	• Caste - High caste men and boys had the best quality food, especially eggs, milk and fish. *Implied that this is less the case with lower caste households.*	Food quality
India (*n* = 7)							
Caldwell, Reddy and Caldwell [51]	1983	In-depth questions and case studies	50% of 4773 population (*n* = 2387)	One large village and eight smaller villages in rural area of southern Karnataka. Individual respondent characteristics not reported.	Daily scrutiny of findings and on-going modification of questions to identify behavioural patterns	• Beliefs about equity - Respondents were reluctant to talk about food allocation. This “demonstrates the existence of some belief in equitable distribution”. Inequity was “as much a matter of poor communication as of deliberate intent”. • Interpersonal relationships – Differential food allocation was in decline due to the strengthening bond between husband and wife.	Food allocation
Daivadanam et al. [59]	2014	Interviews and focus group discussions	17 individuals; 3 groups	Rural areas (one coastal and one non-coastal) Men and women aged between 23 and 75 years, of different religions and socio-economic status. Mostly female heads of household and others involved in dietary decision-making. One group mostly comprised of men.	Modified framework analysis using inductive and deductive reasoning – did not try to fit the data into pre-identified themes.	• Tastes and preferences - women prioritised their own food preferences the least	Allocation of preferred foods
Katona-Apte [39]	1977	In-depth interviews	62 pregnant women or mothers	Two districts from Tamil Nadu. All households had a total income of <200 Indian Rupees per month. All female respondents, and most were pregnant, lactating, or a mother of child under two years.	Analysis method not reported.	• Cultural beliefs about foods – pregnant and lactating women avoided certain foods, and this caused them to have less adequate diets, particularly if there was lack of variety or budget to replace avoided foods with nutritious alternatives	Allocation of specific food types that have different properties
Khan et al. [33]	1987	In-depth interviews and participant observations	20 individuals	One study village from western Uttar Pradesh 20 main female informants (age not reported) from different caste and class groups, and extensive discussions with other villagers, including men.	Analysis method not reported.	• Economic contributions - Respondents said that men should eat more because they earn and provide for the family. The belief that men should be given more food was rarer (3 / 6 respondents) when women earned an income. Some women ate less because they did not have time to eat. Women had less appetite due to fatigue after cooking and serving her family members. • Religious and cultural beliefs - Women “enjoy this spirit of sacrifice for the family”. There was also a belief that pregnant women should eat *ghee* (clarified butter) to give lubrication during birth. The cultural norm of the female cook eating last meant that women eat less. • Status - Women had a religious obligation to fast for the family and for men to have superior status and allocation of food. • Household income - In poor households, the eating order negatively affected women; in wealthy landowning families it did not.	Allocation of food generally, and also of specific food items
Miller [58]	1981	Review of ethnographies	58 studies	Review of many studies from across India.	Meta-analysis	• Interpersonal relationships - Serving food was a way that women show love and affection to their men. Similarly, refusing to eat food was a common method for a man to punish his wife or mother.	Food allocation
Nichols [74]	2016	Semi-structured interviews, and informal conversations and participant observation	81 individuals	Four villages in sub-Himalayan district. Respondents: Government workers, NGO employees, village men, women, and couples. Convenience sampling to include respondents from different class, caste, age, gender and household composition. Plus, national-level NGO representatives from Delhi.	Thematic analysis, by coding themes and intersections between themes	• Labour / physically strenuous economic contributions - women ate the least during planting and harvest seasons when they were working the hardest (and working harder than men) due to a lack of appetite from the exhaustion of the labour	Food allocation
Palriwala [34]	1993	Participant observation	1 village	Sikar district, rural agricultural village with Hindu (85%) and Muslim (15%) castes. Individual participant characteristics not reported.	Not reported	• Cultural beliefs / eating order - youngest daughter in law usually cooks and eats last, leading to less diverse diet as there may be no lentils or vegetables left. • Food scarcity – eating order particularly affected the daughters-in-law during food scarcity. • Economic contributions affect food allocation – income earners are given priority of delicacies and nutrient-rich items like ghee, • Interpersonal relationships – food allocation affected by kinship status, particularly agnation.	Allocation of specific food items
Nepal (*n* = 4)							
Gittelsohn, Thapa and Landman [41]	1997	Key informant interviews, participant observation, unstructured pilot observations, focus group discussions, and structured pile sorts	105 HH	Six rural villages, with a mixture of agricultural and non-agricultural occupations. Men aged 18 to 50 years, and women aged 18 to 50 years, including menstruating, pregnant, lactating, and postpartum women.	Analysis method of qualitative results not reported	• Cultural beliefs - Men were considered the least vulnerable and therefore had the fewest dietary restrictions, unless they were ill. Older people considered vulnerable and believed to require strengthening foods. Some pregnant women mentioned preferentially eating animal products due to ‘craving’. Post-partum women avoided ‘cold’ foods and ‘indigestible’ foods like wheat bread, peanuts, soybeans and corn porridge. They preferentially ate certain ‘hot’ foods like fish and millet roti. Lactating women avoided fresh green leafy vegetables that were perceived as ‘cold’ and believed to cause arthritis, swelling and other illnesses. • Status - Women’s status increased by having children. Before childbearing, young married women had low status and were subtly discouraged from eating special foods like animal products and certain fried foods. Men’s higher status was “recognised in many ways, including household food behaviour”	Allocation of ‘special’ foods
Madjdian and Bras [40]	2016	In-depth interviews	30 individuals	Two Himalayan communities from Humla district. Female respondents (15 Buddhist; 15 Hindu Dalit or Chhetri) of reproductive age (aged 15 to 49 years). Selected respondents who were pregnant or had been pregnant at least once before.	Inductive coding based on a conceptual framework, using bottom-up and top-down coding to allow new themes to emerge.	• Beliefs about ‘fair share’ / Religion - Buddhist households allocated food according to appetite; this was not reported in Hindu households. • Cultural beliefs and food habits - Certain foods believed to cause skin allergies. Eating order was associated with eating less. • Food security - Food insecure households did not adhere to food proscriptions due to a lack of food	Food allocation
Morrison, J. et al. Formative research to inform the development of interventions to tackle low birth weight in the rural plans of Nepal. In preparation.	Unpublished observations	Interviews and focus group discussions	25 women, 2 groups.	One district in *Terai*. 25 young daughters-in-law from marginalised groups living in extended families, one focus group discussion with men, and one with Female Community Health Volunteers who were mothers-in-law. Most (90%) respondents were Hindu. Respondent age not reported	Descriptive content analysis. Data were copied from transcripts into columns of 15 descriptive emergent categories.	• Status - Respondents reported that men ate more because they had higher status and so deserved to. • Interpersonal relationships - Husbands may hide food for their pregnant wives, disrespecting the mother-in-law. • Household structure - Married women who visited or lived at their maternal homes had fewer food restrictions. • Economic contributions – Manual labourers were perceived to deserve more • Cultural food beliefs – pregnant women ate less (fear of full stomach harming the baby) • Household income – no effect of food being bought vs grown on food decisions.	Allocation of food generally, and also of ‘special’ foods
Panter-Brick and Eggerman [64]	1997	Semi-structured interviews	120 heads of household	Population of high and low caste Indo-Nepalese and Tibeto-Burmese ethnic groups from four *Panchayats* in two districts. Sampled households to ensure proportional representation of large and small land-holding farmers Age of respondents not reported.	Analysis method of qualitative results not reported.	• Food shortages / Ethnicity - Indo-Nepalese household used discrimination against women as a coping mechanism during food shortages whereas Tibeto-Burmese households did not.	Food allocation

**Table 3:** Studies with theoretical, hypothetical or general mention of determinants – author, year, determinant and food allocation outcome (*n*=29)

Author	Year	Determinant	IHFA outcome
Bangladesh (*n* = 5)			
Chaudry [70]	1983	Household size	Relative calorie adequacy
Chen, Huq and d’Souza [4]	1981	Relative economic contributions	Allocation of food quantity and quality
Kabeer [44]	1988	Cultural beliefs / serving order	Food allocation
Rizvi [50]	1981	Relative social status	Food allocation
Rizvi [68]	1983	Household wealth (poverty) Household size	Food allocation
India (*n* = 3)			
Cantor and Associates [31]	1973	Relative economic contributions (proxied by body size) Household wealth	Food allocation
Coffey, Khera and Spears [47]	2015	Relative social status	Food allocation
Das Gupta [49]	1996	Relative social status (having sons) Nutrition knowledge	Relative calorie adequacy
Nepal (*n* = 1)			
Gittelsohn, Mookherji and Pelto [42]	1998	Cultural food beliefs Household food insecurity	Food allocation
South Asia (*n* = 2)			
Agarwal [56]	1997	Bargaining power	Food allocation
Appadurai [8]	1981	Relative cultural status / life cycle in the household Bargaining power Interpersonal relationships	Food allocation
International (*n* = 16)			
DeRose, Das and Millman [14]	2000	Relative social status Decision-making Nutrition knowledge	Calorie and food allocation
Haddad and Kanbur [25]	1990	Control over income Food insecurity	Calorie and food allocation
Haddad et al. [11]	1996	Decision-making (identify of decision-maker) Food insecurity	Food allocation
Haddad [54]	1999	Control over income Food insecurity	Food allocation
Kumar [26]	1983	Decision-making	Food allocation
Messer [48]	1997	Relative social status (the traditional role and perceptions of women) Beliefs about fairness	Food allocation
Pinstrup-Andersen [15]	1983	Nutritional need Preferences Decision-making Household income	Food allocation
Wheeler [12]	1991	Relative economic contributions Beliefs about fairness Bargaining power	Allocation of nutrient-rich foods
Carloni [53]	1981	Decision-making Social mobility / participation in shopping	Food allocation
Hartog [28]	1972	Economic contributions Cultural beliefs Social status Interpersonal relationships	Food allocation
De Schutter [52]	2013	Beliefs about fairness Control over food production or purchasing Food insecurity	Food allocation
Den Hartog [43]	2006	Religious beliefs Beliefs about fairness	Food allocation
Gunewardena [60]	2014	Food insecurity	Food allocation
Pelto [46]	1984	Social status (in relation to modernisation and urbanisation)	Food allocation
Ramachandran [55]	2007	Decision-making / control over income Bargaining power Food insecurity Household composition (nuclear vs joint households)	Food allocation
Van Esterik [29]	1985	Economic contributions Overlap between cultural beliefs during pregnancy, social status, and poverty Social mobility Interpersonal relationships Household size (number of senior women) Religion Food availability	Food allocation
No countries mentioned (*n* = 2)			
Doss [24]	1996	Relative economic contributions Bargaining power	Food allocation
Hamburg et al. [57]	2014	Interpersonal relationships	Food sharing

### Quality assessment of selected papers

There was limited empirical evidence, a diverse range of methods used, heterogeneity in the outcome measure, a high proportion of anecdotal results, and limited methodological detail. This meant that it was not possible to conduct a meta-analysis of any key determinants. Quality assessments for quantitative and qualitative results are given in Tables 4 and 5 respectively.

**Table 4:** Quality assessment of quantitative results (*n* = 16) using an adapted Downs and Black checklist

Study quality	No	Unable to determine	Yes	
	*n*	*n*	*n*	(%)
Is the hypothesis or aim of the study clearly described?	0	NA	16	(100)
Are the outcomes described in the Introduction or Methods?	1	NA	15	(94)
Are the characteristics of the respondents described?	6	NA	10	(63)
Are the determinants of interest described?	2	NA	14	(88)
Are the distributions of principal confounders described?	6	NA	10	(63)
Are the main findings of the study clearly described?	1	NA	15	(94)
Does the study provide estimates of random variability?	9	NA	7	(44)
Have probability values (not cutoffs) been reported?	14	NA	2	(13)
Validity, bias and confounding				
Was the sample representative of the population?	1	7	8	(50)
Were the respondents representative of the population?	0	14	2	(13)
Were the statistical tests appropriate?	4	0	12	(75)
Were the main outcome measures used valid and reliable?	0	3	13	(81)
Was there adequate adjustment for confounding?	8	2	6	(38)
Were losses of respondents taken into account?	3	11	2	(13)

**Table 5:** Quality assessment of qualitative results (*n* = 15) using Critical Appraisal Skills Programme (CASP) checklist

Critical Appraisal Skills Programme (CASP) quality indicator	No	Unable to determine	Yes	
	*n*	*n*	*n*	(%)
Was there a clear statement of the aims of the research (the goal, importance, and aims)?	0	0	15	(100)
Is a qualitative methodology appropriate?	0	0	15	(100)
Was the research design justified as appropriate to address the aims of the research?	0	7	8	(53)
Was the recruitment strategy justified as being appropriate to the aims of the research (how and why respondents were sampled, or discussions of non-response)?	0	7	8	(53)
Were the data collected in a way that addressed the research issue (detail and justification of methods, issues of data saturation)?	0	9	6	(40)
Has the relationship between researcher and participants been adequately considered?	0	9	6	(40)
Have ethical issues been considered (informed consent and ethical approval)?	0	13	2	(13)
Was the data analysis sufficiently rigorous?	0	10	5	(33)
Is there a clear statement of findings?	1	0	14	(93)

Quality assessments showed that the results were limited by the representativeness of the samples, and methods for quantifying and addressing non-response. Quantitative results rarely gave exact *p*-values (in many cases there was no statistical test) and few qualitative studies discussed the potential influence of the interviewer on the respondents’ answers, the rationale for their sampling methods, or their analysis techniques.

### Framework of determinants of intra-household food allocation

**Fig. 2:**
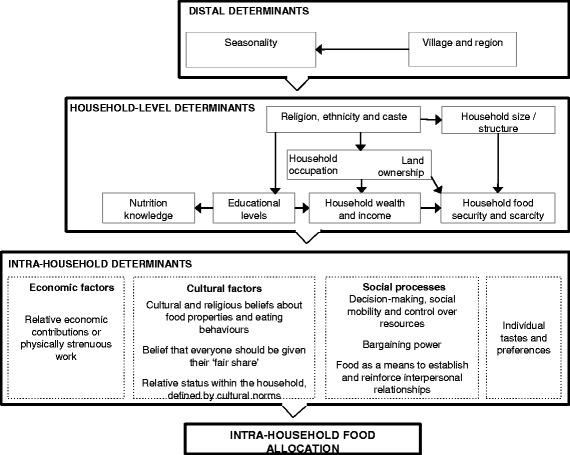
Determinants of intra-household food allocation and hypothesised hierarchical structure

Eighteen determinants emerged from the thematic analysis and are illustrated in a framework in Fig. 2. The framework also gives intuitive (rather than evidence-based) hierarchy and linkages between determinants. These linkages are not given at the intra-household level, where categorisation of a complex reality into boxes becomes particularly difficult as determinants overlap, complement or compete with one another.

The findings are narratively summarised by determinant, starting at the intra-household level, then the household level, and finally the distal determinants.

### Intra-household level determinants

#### Relative economic contributions or physically strenuous work

Many theoretical or anecdotal studies suggested that selective investment in economically productive members (typically adult men) was a rational household survival strategy in the South Asian context of predominantly manual farming labour and economic returns to health [12, 24–29]. However, these gender differences were hypothesised to differ by wealth status, with poorer households being more reliant on women’s economic productivity (and so more equitable) than less poor households [4].

Three quantitative studies corroborated this food-health-income link, although none directly measured the effect of relative income on food allocation. Rathnayake and Weerahewa [30] found that mothers’ incomes were positively associated with their own relative calorie allocations, but the authors did not adjust for total household income. Using body size as a proxy for economic capacity, Cantor and Associates [31] found a positive association between body size and relative food allocation, although it is not known whether the authors adjusted for the higher energy requirements associated with heavier people. Pitt et al. [32] found that men’s health ‘endowments’ (pre-existing health status) determined their allocations of food, whereas women’s did not; a 10% increase in health endowment was associated with a 6.8% increase in calorie intake for men but only one tenth of that for women [32]. These differences were posited to reflect gender differences in economic returns to nutritional investment.

The food-health-income linkage was supported by qualitative studies that reported respondents’ beliefs that income earners deserved to be allocated more food [33, 34], but this was often conflated with beliefs about physiological requirements, particularly for manual labourers ([35] and Morrison J, Dulal S, Harris-Fry H, Basnet M, Sharma N, Shrestha B, Manandhar DS, Costello A, Osrin D, Saville N: Formative qualitative research to develop community-based interventions addressing low birth weight in the plains of Nepal. Working draft, in preparation). One study also reported beliefs that elderly people should be allocated favoured foods to acknowledge their ‘past contributions’ to the household [36], and that men required more than women because men’s work was more physically strenuous than women’s home-based work [36]. Conversely, Hartog [28] anecdotally suggested that the allocation of food according to economic contributions was no longer justified because of the increased mechanisation of agricultural work, and Aurino [37] supported this empirically. There was no association between gender differences in workload (with 15-year old girls working significantly more than 15-year old boys) or frequency of exercise and the gender differences in dietary diversity.

#### Cultural and religious beliefs about food properties and eating behaviours

Sixteen studies described cultural beliefs about food properties and eating behaviours as a determinant of food allocation, typically showing how these beliefs caused women to receive comparatively less.

Some foods were believed to be ‘unhealthy’, ‘strengthening’ or ‘digestible’, and therefore deliberately avoided or selected by vulnerable people, such as elderly people, or pregnant or lactating women [28]. Short-term ‘transitory’ states, such as menstruation and illness that are perceived to make a person ritually unclean (‘*jutho’* (Nepalese))*,* were reported to cause inequity in food allocation [9, 38]. Certain fruits and vegetables were considered unhealthy for post-partum or lactating women [9], and other foods were believed to cause illness [39] or skin allergies [40] in the breastfeeding child. Elderly people were believed to require soft ‘digestible’ [9] and ‘strengthening’ foods [39, 41]. Pregnant women were reported to require *ghee* (clarified butter), to facilitate an easier, lubricated birth [33], but avoid certain foods that cause illness, indigestion, fits, delirium, or large babies (and therefore difficult labour) [39] and eat less because of a belief that a full stomach would harm the baby (Morrison J, Dulal S, Harris-Fry H, Basnet M, Sharma N, Shrestha B, Manandhar DS, Costello A, Osrin D, Saville N: Formative qualitative research to develop community-based interventions addressing low birth weight in the plains of Nepal. Working draft, in preparation). Another phase condition is puberty; according to the ‘pubertal hypothesis’ households might change intra-household food allocation in favour of adolescents going through pubertal growth spurts, however this was not supported empirically [37].

The categorisation of foods as heating, neutral or cooling was also linked to differential food allocation [9]. Examples of ‘heating’ foods included wheat, fish, millet, and milk*,* and ‘cooling’ foods included yogurt, fruits, green beans, gourds, and rice [41]. Again, these categories were related to the condition of the individual; for instance, lactating women avoided cooling foods [41]. Some authors noted that these restrictions caused an overall reduction in women’s dietary intake [41] perhaps due to insufficient household budget for nutritious alternatives [39]. The overlap of women being in the ‘phase condition’ of being pregnant, having low social status, and extreme poverty, was hypothesised as a cause of inequitable allocation of food towards pregnant women [29], whereas another study found that food insecure households did not adhere to food proscriptions due to a lack of food [40].

Cultural beliefs about the status or value of foods were also linked to food allocation [42]. Some people were allocated or ‘channelled’ specific foods, and these foods were often high status and expensive. For example, *ghee* (a high status food) was often only given to men [9] (perhaps with the exception of pregnant women, as documented earlier). Food ‘channelling’ was associated with significantly higher food quantities, so those who were given special foods were also given more food in total [9].

Religious beliefs may affect the distribution of food. For example, beliefs about the meaning of food, such as the act of eating being considered a form of worship in Islam, was suggested as a determinant of food allocation, although the direction of effects was not specified [43]. Fasting caused women to be allocated comparatively less food, because women fasted more frequently and strictly than men [27, 33].

Eating order was another key factor in food eating and allocation behaviours. Daughters-in-law, often young, newly married women, tended to serve themselves last (to show deference and ensure the wellbeing of male members [44]) and this was associated with eating less [9, 33, 40] and also lower quality diets than others [34, 45]. Gittelsohn [9] found that late serving order at meal times, being the food server, and not having a second helping were all significantly negatively associated with the quantity of food consumed. Two authors suggested that the negative effects of serving order were caused by limited food availability, because women tended to ensure everyone else had enough before serving themselves the remainder [9, 44].

#### Relative social status within the household, according to cultural norms

High social status and perceived deservedness of household members was reported by 14 studies as a determinant of intra-household food allocation, although no studies quantified the effect empirically. Some mentioned status in general terms, without ascribing high or low status to particular household members but suggesting that people with lower status would receive less food and less preferred foods [12, 14, 28, 29, 46], whereas others reported that men had higher status and therefore were allocated more food [9, 41, 47]. Pelto [46] noted that modernisation and increasing urbanisation was reducing the effect of status on food allocation.

Women’s identity as being lower status, frugal, modest, and subservient was described as a determinant of food allocation that favoured men but was often internalised by women [33, 48]. This identity was also interlinked with perceptions of body image, that also led women to eat comparatively less [48].

In addition to differences between genders, food allocation was also determined by differences in social rankings among women within households [47], hierarchy within the patriline [34], and variations within individuals over time. Women received more food as their status increased with age [9], by having children [41], and, particularly, by having sons [49]. In contrast, Rizvi [50] anecdotally suggested that increases in female status over time did not affect food allocation.

#### Belief that everyone should be given their ‘fair share’

Overlapping these beliefs about food properties and status were locally-held beliefs about fairness, and different definitions of what ‘fair’ means [48]. An Indian study concluded that beliefs about fairness affected food allocation because the respondents were reluctant to discuss disparities in food allocation [51]. Abdullah [36] also indicated this, by reporting a respondent’s description of a mother-in-law as a “bad woman” (p112) if she did not give her daughter-in-law special foods that others received, and Madjdian and Bras reported that Buddhist households allocated food according to appetite [40]. Pitt et al. reported that the tendency for households to equalise food allocation, rather than allocate food in an income-maximising way, was a reflection of households’ aversions to inequity [32]. Pinstrup-Andersen also hypothesised that perceived nutritional need determined food allocation (arguably a definition of fairness) [15]. Conversely, three others reported an ideological belief that men deserved to be given more than women [33, 43, 52].

#### Decision-making, social mobility, and control over resources

The ten studies on decision-making, control over resources, social mobility, or identity of the cook were all anecdotal or theoretical. Few specified who the decision-maker was or who would benefit from the decisions [15, 26, 53]. Haddad et al. noted that female decision-makers may be more likely than male decision-makers to allocate food in a way that maximised the household’s nutritional outcomes [11], and other work suggested that women’s control over income [25, 54, 55] (or production or purchasing [52]) would affect food allocation in favour of women. However, decision-making in nuclear households was also a risk factor for inequitable allocation towards women, as female decision-makers were responsible for ensuring that everyone else was sufficiently fed [55].

Related to this was different household members’ social mobility, and freedom to go food shopping or access food outside of the home [29, 53]. Women were described as having less social mobility and this was linked to lower food allocations [53]. However, it was also suggested that there may be less inequity against women than expected because food allocation occurs within the household, which, compared with the public sphere, is a domain in which women traditionally have more control [14].

#### Bargaining power

Related to this, many studies suggested that ‘bargaining power’ was an important determinant of food allocation. Some described a general linkage [55], while others described various microeconomic theories whereby individuals rationally aim to maximise their utility by bargaining over household resources such as food [12, 24, 56]. The strength of a person’s bargaining power is determined by their ‘fallback position’ (the utility that the individual would achieve if cooperation with other household members fails). Bargaining power may be predicted by the size of dowry for women [55], social norms that determine how goods such as food can be exchanged or used, and /or perceived requirements [56]. Naved also indicated that bargaining power might affect food allocation, with specific reference to micronutrients [35]. Appadurai described the process of bargaining with food, particularly in reference to daughters-in-law feeling resentment about their subservient role [8]. Daughters-in-law were reported to communicate resentment by being reluctant to cook, destroying food, or making small signs of discontent when serving food [8].

#### Food as a means to establish and reinforce interpersonal relationships

A study on the psychology of feeding found that food serving may be determined by the emotional state of the server and recipient, and that feeding was done to build relationships [57]. Serving and eating food was reported as a key determinant of intra-household food allocation [34] and a means to indicate rank [8], show disrespect or displeasure ([58] and Morrison J, Dulal S, Harris-Fry H, Basnet M, Sharma N, Shrestha B, Manandhar DS, Costello A, Osrin D, Saville N: Formative qualitative research to develop community-based interventions addressing low birth weight in the plains of Nepal. Working draft, in preparation), punish or reward people [29], or express love and strengthen relationships [28]. Two studies suggested that a strong bond between husband and wife might reduce the inequity against the wife that would otherwise be imposed by the mother-in-law ([51] and Morrison J, Dulal S, Harris-Fry H, Basnet M, Sharma N, Shrestha B, Manandhar DS, Costello A, Osrin D, Saville N: Formative qualitative research to develop community-based interventions addressing low birth weight in the plains of Nepal. Working draft, in preparation).

#### Individual tastes and preferences

One study [15] reported a general hypothesis that food preferences determine allocations. One study found that women were allocated the less-preferred foods [35], and another found that women prioritised their own food preferences the least [59]. A new vegetable production program, which had increased the availability of less popular vegetables, had caused an increase in women’s consumption of those vegetables [35].

### Household-level determinants

#### Food insecurity and scarcity

Evidence on the effect of household food security was mixed. Seven studies provided a hypothetical link between food insecurity and food allocation [11, 25, 42, 52–54, 60].

Most studies suggested that women were more sensitive to changes in food availability, and acted as a buffer for the household in food insecure conditions [40, 52, 55], particularly the youngest daughter-in-law [34]. Household calorie adequacy was a better predictor of calorie adequacy for women than for men [61], years of higher rice yields were linked to higher equity in calorie allocation [62], and women were more sensitive to food price changes (had higher food price elasticity) than men [63]. Although one study found no overall effect of food insecurity, there were differential effects between villages, and in food-scarce months landowning households favoured men at the expense of women while labourer households favoured adults at the expense of children [27]. Another study found some ethnic variability in these effects, with Indo-Nepalese household using discrimination against women as a coping mechanism during food shortages and Tibeto-Burmese households not changing their food distribution patterns in this way [64].

In contrast, some studies found opposing or no effects. Naved found that increased food *availability* did not affect food allocation patterns, but food *scarcity* led to men being less likely to have sufficient food than women [35]. Abdullah also found women’s proportion of men’s intakes increased from 81 to 90% between food secure and food short seasons, in poor households [36]. A later analysis by the same authors found no significant effect after adjusting for differential requirements [65], and another also found no effect of scarcity on calorie allocation [66].

#### Household wealth and income

Evidence was mixed on the effect of household wealth and income. Three quantitative studies reported no effect [31, 38, 67], while Pinstrup-Andersen’s [15] theoretical study hypothesised that there was an effect without specifying the direction. Two qualitative studies reported higher inequity in poorer households [33, 68], but a qualitative study reported that the source of food, whether bought or grown, had little effect on food decisions (Morrison J, Dulal S, Harris-Fry H, Basnet M, Sharma N, Shrestha B, Manandhar DS, Costello A, Osrin D, Saville N: Formative qualitative research to develop community-based interventions addressing low birth weight in the plains of Nepal. Working draft, in preparation).

#### Educational levels

Households with more educated household heads allocated less meat to women than men [69], whereas another two studies on female education showed no association with food allocation [30, 37].

#### Nutrition knowledge

Although studies linked food beliefs and food allocation, no studies measured the association between dietary knowledge and food allocation. Das Gupta suggested that lack of knowledge did not explain the comparatively inadequate intakes of pregnant and breastfeeding women in India [49], whereas others suggested the opposite [14, 38].

#### Household occupation

One study found that the greatest gender inequity occurred in agricultural labourer households, compared with market-based, subsistence farmers or non-agricultural households [62]. Another found that farm work and household chores were associated with consuming fewer snacks, and that all three (farm work, household chores, and snacks in a combined score) were significantly higher for women than men [45]. In contrast, two studies found no effect of being an agricultural labourer household [69] or household occupation [67] on food allocation. However, if the household head was a farmer, then milk was allocated more equitably and meat less so [69].

#### Land ownership

Only Bouis and Novenario-Reese mentioned the effect of land ownership, finding that landowning households were less equitable with their allocation of eggs but more equitable with fish [69].

#### Household size and structure

The effect of household size was also mixed. Van Esterik suggested that the number of senior women in a household would affect food allocation patterns [29], while Rizvi suggested that household size affected intakes of individuals, but not allocation patterns [68]. Rathnayake and Weerahewa reported a significant positive effect of household size on mothers’ relative calorie allocations in Sri Lanka [30], and a study from Pakistan also found higher intakes for the household head, pregnant women, and children under five years, relative to average household intakes, in larger households [38]. Conversely, another study showed that large household size (more than eight children) was associated with significantly higher male than female calorie adequacy [70].

(Morrison J, Dulal S, Harris-Fry H, Basnet M, Sharma N, Shrestha B, Manandhar DS, Costello A, Osrin D, Saville N: Formative qualitative research to develop community-based interventions addressing low birth weight in the plains of Nepal. Working draft, in preparation) reported a trend for food-related rules to be relaxed when women returned to their parental homes, where they had fewer food restrictions. This was supported by Abdullah [36], who found that women were given less or fewer special foods in the marital home, and so women (or adolescent, unmarried girls) received special treatment when at their parental homes. Comparing joint and nuclear households, Ramachandran reported that women were allocated comparatively less in nuclear households, where the women had the responsibility of feeding everyone; whereas, in joint households women were given more because their mother-in-law adopted the role of food planning and distribution [55].

#### Religion, ethnicity and caste

Evidence on the effects of religion, ethnicity and caste was limited and mixed. Van Esterik [29] hypothesised that religion was a determinant of food allocation, particularly through religious influence on food classifications systems. There were no differences between Buddhists and Christians in food allocation [67], but Buddhist families were more equitable than Hindu households [40]. Rathnayake and Weerahewa found no effect of ethnicity on relative calorie allocations [30], whereas Panter-Brick and Eggerman found that food shortages caused discrimination against women during food shortages among Indo-Nepalese households but not among Tibeto-Burmese households [64]. No studies explicitly described the effect of caste, but two studies implied that inequity would be greater in high caste groups [63, 71].

### Distal determinants

#### Seasonality

Two quantitative studies relating to seasonality found contradictory, non-significant and unexplained effects on intra-household food allocation. In Bangladesh, Tetens et al. found more equity in food allocation during peak agricultural production seasons [72], whereas in India Chakrabarty found less equity in cereal consumption in the peak season [73]. A qualitative study from India found that women ate the least during planting and harvest seasons when they were working the hardest (and working harder than men) due to a lack of appetite from the exhaustion [74], and a seasonal calendar from Bangladesh showed that women were allocated fewer eggs and fish than men, particularly during food insecure months [75]. An anecdotal study suggested that predictable seasonal variation was unlikely to affect food allocation patterns because households would have coping strategies for predictable variations in food availability [29]. However, if this shortage overlapped with periods of more pregnancies or more female agricultural labour inputs, then women would have less adequate diets than other household members [29].

#### Village and region

Regional differences in intra-household food allocation were varied. Basu et al. found no differences between urban and rural areas among the Lechpas ethnic group in India [67], whereas a study from Pakistan found small but significant regional variation in micronutrient allocation [38], with higher dietary adequacy for men in certain regions and lower adequacy for pregnant or lactating women in others. The authors suggested that this may have been a result of regional differences in cultural attitudes and beliefs regarding pregnancy, but did not describe these attitudes in detail. Harriss-White also found differences in inequity between four different villages in India, which she hypothesised may have been due to differences in land ownership and methods of agricultural production [27].

## Discussion

Although we searched for literature across South Asia, studies were mostly from Bangladesh, India, and Nepal, with few from Islamic Republic of Iran, Pakistan, and Sri Lanka, and none from Afghanistan, Maldives or Bhutan. We identified eighteen determinants of intra-household food allocation, many of which were specific to the South Asian context and centred on interlinked aspects of poverty, cultural beliefs, and power. The review suggests that the most equitable households are low caste households [63] in which women earn an income [30] and there is low educational status of the household head [69] (indicative of lower socioeconomic status) but that equitable households will also be food secure [61] (indicative of higher socioeconomic status).

We hypothesise that these seemingly conflicting effects could be reconciled in a non-linear relationship between socioeconomic status and inequity. During acute or unexpected food shortages, households selectively invest limited calories in men because they have more labour opportunities [62, 63]. For example, recent unexpected shocks may include the 2015 Nepalese earthquakes [76]. As food security increases and food shortages become more predictable, households become more equitable [65, 72, 73]. Low caste households are often described as more egalitarian [71], partly because low caste men do not inherit land, [5] but also because women have comparatively higher economic contributions [73]. The positive effect of individuals’ relative economic contributions on their shares of household food is also supported by evidence from the Philippines [77] and China [78]. At higher levels of socioeconomic status, inequity may increase again [27]. This is supported by the finding that women from landowning Indian households are thinner than in landless households [45]. Inequity amongst higher socioeconomic groups may be characterised by preferential “channelling” of high status, often micronutrient-rich luxury foods such as meat or dairy [9], rather than by unequal allocation of staples that may occur in the poorest groups [62]. If true, this channelling could result in social rather than nutritional inequity, or perhaps inequity of micronutrients rather than calorie allocation. This may explain the surprising negative gradient in the prevalence of anaemia in women with increasing wealth in Nepal (anaemia: 32% in the lowest wealth quintile, and 49% in the middle quintile) [79], despite the relatively high cost of micronutrients required to reduce anaemia. This inequity may decline in households where women are highly educated and ‘modern’, as they may have the most knowledge of dietary requirements (to counteract discriminatory cultural food practices) and these households may be less socially conservative and restrictive towards women. Although no studies have provided evidence to support this directly, the prevalence of anaemia in Nepal does fall to 36% in the highest wealth quintile [79].

Any socioeconomic level, dynamic intra-household factors (such as bargaining, preferences and interpersonal relationships) will introduce variance within these trends, while cultural norms and food practices (that have wide local variation but are slow to change [40]) may introduce variance and also determine the strength of association between socioeconomic status on food allocation.

### Limitations of the study, and future work

This study benefitted from a systematic literature search but the results and conclusions are limited by the lack of recent evidence. Given the rapid changes in labour migration, engagement in non-farm work, and mechanisation of farm work [80], the full framework should be tested with recent data. Another limitation is that most studies came from Bangladesh, India and Nepal, so the results may be less or not valid for other South Asian countries that did not have any or many studies (particularly Afghanistan, Bhutan, Maldives, Iran, and Pakistan). Even within the results from India, Bangladesh and Nepal, there is wide heterogeneity in culture, beliefs, and institutions, making generalisations difficult. Many results arose from snowballing, indicating poor indexing of multi-disciplinary evidence, so the included studies may be subject to citation bias.

Most papers considered only a few determinants in their analyses and did not control for possible confounders, so the pathways in the framework cannot be disentangled. For example, we cannot tell whether papers that tested the effects of seasonality or wealth on food allocation would have found the same results after controlling for household food security. Similarly, the overlap of cultural norms, social status, income earning, bargaining power, and participation in decision-making, means that the effects of these intra-household determinants cannot be distinguished. We expect that these intra-household determinants co-exist, sometimes reinforcing and other times opposing each other.

To truly understand these intra-household processes, longitudinal mixed methods are required to examine each possible determinant at different stages in the household life cycle (adolescents, newlyweds, senior members, elderly), with different phase conditions (during puberty, illness, menstruation, pregnancy, post-partum, and breastfeeding), at different points in the food acquisition–preparation–distribution pathway, in different socioeconomic groups, in different regions and in different seasons. Given the breadth of this evidence gap, research to inform nutrition program design could particularly focus on the determinants of food allocation that may be amenable to change, such as food security, women’s employment, or nutrition knowledge. Most studies focused on energy or food quantity, so more research is needed on the allocation of micronutrient-rich foods [27]. Other next steps could link the evidence on factors affecting the determinants in this framework, such as predictors of food choice, bargaining power, and food security.

### Implications of the findings: what does this mean for nutrition interventions?

The findings indicate that women are disadvantaged in the allocation of food, and that women’s nutrition outcomes could be improved through changes in intra-household food allocation patterns. In particular, pregnant women tend to receive lower relative allocations, and this has important nutritional implications because nutrition during pregnancy is associated with maternal health outcomes, intra-uterine growth retardation, child health outcomes, and is a key point in the intergenerational cycle of undernutrition [81]. As such, interventions may need to particularly prioritise pregnant women.

By predicting the distribution of transfers at all levels of socioeconomic status, social transfer programs could be designed to ensure that the intended recipients receive the transfers. For example, programs delivering transfers in emergency contexts may need to ensure that transfers are large enough [82], so that transfers will reach less economically productive household members. Poorer households might require additional resources such as food or cash transfers to improve the nutritional status of women. Low caste households may be more likely to share these transfers equitably, and so additional intervention components (such as behaviour change communication) to ensure the transfers reach target recipients may only be required if the programmer intends to disproportionately target women (or pregnant women).

In contrast, transfers to high caste or better-off groups may need to include a behaviour change component to ensure that transfers reach women. Alternatively, programs delivering transfers to high caste groups could provide lower status, less desirable goods, such as flour rather than rice [83], to ensure that transfers will be preferentially distributed to lower status household members. This leads to a wider discussion on programmatic objectives, and whether programs intend to challenge patriarchal norms to empower women and improve nutrition, or to work within patriarchy to achieve optimal nutritional outcomes. Furthermore, high caste better-off households that can already afford more micronutrient-rich foods may not need more resources to improve maternal nutrition – behaviour change interventions may be sufficient.

Alternative, or complementary, approaches to improve intra-household equity in food allocation may include: interventions that provide income-generating activities for women, women’s groups to increase social mobility and bargaining power or otherwise empower women, or agricultural programs to improve food security.

## Conclusions

There are many possible household-level and intra-household determinants of intra-household food allocation, but evidence is out-dated and not comprehensive. Local context and variation in social hierarchies makes generalised conclusions difficult. Programs delivering social transfers may find differential intra-household distribution of transfers in different socioeconomic groups. Programs affecting determinants that are amenable to change, such as household food security, bargaining power, and gender-specific labour opportunities, may cause changes in intra-household food allocation patterns.
